# Improving drug-drug interaction prediction via in-context learning and judging with large language models

**DOI:** 10.3389/fphar.2025.1589788

**Published:** 2025-06-02

**Authors:** He Qi, Xiaoqiang Li, Chengcheng Zhang, Tianyi Zhao

**Affiliations:** ^1^ School of Medicine and Health, Harbin Institute of Technology, Harbin, China; ^2^ Center for Drug Evaluation and Inspection for Heilongjiang Province, Harbin, China; ^3^ Suzhou Institute of Biomedical Engineering and Technology, Chinese Academy of Sciences, Suzhou, China; ^4^ Faculty of Computing, Harbin Institute of Technology, Harbin, China; ^5^ Harbin Institute of Technology Zhengzhou Research Institute, Zhengzhou, China

**Keywords:** large language models, drug-drug interactions, in-context learning, zero-shot, few-shot

## Abstract

**Introduction:**

Large Language Models (LLMs), recognized for their advanced capabilities in natural language processing, have been successfully employed across various domains. However, their effectiveness in addressing challenges related to drug discovery has yet to be fully elucidated.

**Methods:**

In this paper, we propose a novel LLM based method for drug-drug interaction (DDI) prediction, named DDI-JUDGE, achieved through the integration of judging and ICL prompts. The proposed method outperforms existing LLM approaches, demonstrating the potential of LLMs for predicting DDIs. We introduce a novel in-context learning (ICL) prompt paradigm that selects high-similarity samples as positive and negative prompts, enabling the model to effectively learn and generalize knowledge. Additionally, we present an ICL-based prompt template that structures inputs, prediction tasks, relevant factors, and examples, leveraging the pre-trained knowledge and contextual understanding of LLMs to enhance DDI prediction capabilities. To further refine predictions, we employ GPT-4 as a discriminator to assess the relevance of predictions generated by multiple LLMs.

**Results:**

DDI-JUDGE achieves the best performance among all models in both zero-shot and few-shot settings, with an AUC of 0.642/0.788 and AUPR of 0.629/0.801, respectively. These results demonstrate its superior predictive capability and robustness across different learning scenarios.

**Development:**

These findings highlight the potential of LLMs in advancing drug discovery through more effective DDI prediction. The modular prompt structure, combined with ensemble reasoning, offers a scalable framework for knowledge-intensive biomedical applications. The code for DDI-JUDGE is available at https://github.com/zcc1203/ddi-judge.

## 1 Introduction

Polypharmacy, or the simultaneous use of multiple drugs, is common in the treatment of patients with various diseases (van Roon et al., 2005). However, it can lead to adverse drug reactions (DDIs) due to drug–drug interactions. DDIs are responsible for 30% of all reported adverse drug reactions, significantly impacting patient safety, morbidity, mortality, and healthcare costs (Ryu et al., 2018). Given the complexity of diseases and the limitations of single-drug therapies, combination therapies have the potential to improve efficacy, but they also increase the risk of unintended interactions (Deng et al., 2020). Therefore, accurate DDI prediction is crucial for improving treatment outcomes and minimizing adverse effects. Although DDI research has become a major focus, the identification of DDIs remains challenging due to limited clinical trial resources and the rapid growth of biomedical data.

Current state-of-the-art DDI prediction methods include traditional machine learning and deep learning approaches. Among these, deep learning methods leverage technologies such as deep neural networks (DNNs) (Sze et al., 2017), convolutional neural networks (CNNs) (Alzubaidi et al., 2021), graph neural networks (GNNs) (Wu et al., 2020) and transformer (Vaswani, 2017), achieving remarkable performance. However, these methods often perform poorly in zero-shot scenarios and exhibit limited capability in learning from large-scale, multi-source data integration.

Large language models (LLMs), exemplified by architectures such as GPT-4 (Achiam et al., 2023), Claude (Ryu et al., 2018; Bai et al., 2022), llama (Touvron et al., 2023a), and Mistral (Jiang et al., 2023), have demonstrated transformative capabilities in general-domain tasks through their massive parameter spaces, self-supervised pretraining frameworks, and attention-based neural architectures. While LLMs demonstrate exceptional performance in general tasks, their capabilities in specialized application domains remain significantly constrained.

In the field of drug discovery, LLM have demonstrated significant potential in several directions, including the integration of multi-source data (Wan et al., 2024), the design of downstream tasks (Guo et al., 2023), and the optimization of prompting strategies for specific applications (Guo et al., 2024). These advancements have enabled LLMs to perform tasks such as molecular property prediction and molecular translation. However, critical challenges persist in applying LLMs to DDI prediction: 1) the scarcity of high-quality, annotated DDI datasets due to expensive experimental validation; 2) poor generalizability under zero-shot learning conditions, particularly for rare interaction types; 3) ineffective fusion of heterogeneous data modalities spanning molecular structures, pharmacological pathways, and clinical context.

To overcome these limitations, we introduced the DDI-JUDGE model, which employs in-Context Learning (ICL) to propose a prompt paradigm tailored for DDI tasks and leverages a judge to integrate the predictive capabilities of multiple LLMs.

The main contributions of this paper are as follows:

1) We propose a DDI prediction method based on large language models enhanced by judging and in-context learning, named DDI-JUDGE.
2) We propose a novel ICL prompt paradigm for DDI prediction, employing cosine similarity-based exemplar retrieval for in-context learning and coupling it with an ensemble discriminator module, such as GPT-4, that strategically aggregates predictions from heterogeneous LLMs through confidence-weighted voting, thereby improving robustness against model bias.
3) The effectiveness of our method has been demonstrated through comprehensive experiments in both zero-shot and few-shot scenarios, outperforming other LLM methods.

The structure of this paper is as follows: The Related Work section provides a brief review focusing on methods of DDI prediction. The Methods section offers a detailed description of the proposed DDI-JUDGE method. The Experiments and Results section presents the experimental setup and analyzes the results. Finally, the Conclusion section summarizes the key points and discusses potential directions for future research.

## 2 Related work

### 2.1 Methods of drug-drug interactions prediction

DDI prediction has been an essential area of research due to its critical implications for rational drug use, enhancing therapeutic efficacy, and minimizing adverse drug reactions. Numerous computational models, including traditional machine learning and deep learning approaches, have been developed for DDI prediction.

Traditional machine learning models predict DDIs by leveraging features such as drug similarity, protein-protein interaction networks, and drug phenotypic profiles. For instance, Bayesian models calculate interaction scores based on protein networks and drug phenotype similarity (Huang et al., 2013). Label propagation-based models (Zhang et al., 2015) integrate drug side effects and chemical structure data, while probabilistic frameworks, such as the collective soft logic model (Sridhar et al., 2016) rely on multi-source similarity features. Additionally, manifold regularization and matrix factorization approaches, like DDINMF (Yu et al., 2018) and TMFUF (Shi et al., 2018), enhance predictions by incorporating semi-nonnegative matrix decomposition and manifold structures.

Deep learning methods have significantly enhanced DDI prediction by enabling complex feature extraction and multi-source data integration. Models like DDIMDL (Deng et al., 2020) and CNN-DDI (Zhang et al., 2022) employ deep neural networks (DNNs) and CNNs, respectively, to calculate interaction probabilities using drug similarity matrices. Graph-based methods, such as SSI-DDI (Nyamabo et al., 2021), convert SMILES strings into molecular graphs and utilize graph attention networks (GATs) to extract substructure representations. Tensor-based approaches like STNN-DDI (Yu et al., 2022) employ tensor factorization to predict interaction types. Network-based methods have further refined DDI prediction by incorporating multi-relation and heterogeneous data. For instance, META-DDIE (Deng et al., 2022) combines frequent substructure mining and neural encoding for DDI type prediction, while DANN-DDI (Liu et al., 2022) employs attention mechanisms to generate comprehensive drug embeddings from heterogeneous networks. MRCGNN (Xiong et al., 2023) utilizes multi-relation DDI event graphs with relational graph convolutional networks for feature extraction. Similarly, SubGE-DDI (Shi et al., 2024) integrates substructure representations from molecular graphs with attention-based mechanisms to improve prediction accuracy. Furthermore, KGE-UNIT (Zhang et al., 2024) enhances DDI prediction performance by multi-task learning. These network-driven and hybrid approaches offer significant improvements by combining molecular, structural, and contextual data in highly integrated frameworks. However, the current method has insufficient learning ability for massive multi-source data and cannot adapt well to the zero-shot scenario.

### 2.2 Large language models for drug discovery

LLM have shown significant potential in advancing molecular science by bridging textual information and molecular data, which has facilitated applications such as molecule retrieval, reaction prediction, and drug discovery. Recent studies, such as Text2Mol (Edwards et al., 2021), Molxpt (Liu Z. et al., 2023), and Mol-Instructions (Fang et al., 2023), have established connections between molecular structures and textual descriptions, enhancing tasks such as molecule editing, annotation, and retrosynthesis. In drug development, Y-Mol (Ma et al., 2024) and DrugReAlign (Wei et al., 2024) demonstrate the versatility of LLMs in tackling complex tasks. Y-Mol offers a biomedical knowledge-guided approach for virtual screening, property prediction, and drug interaction prediction, enhancing domain-specific reasoning. Meanwhile, DrugReAlign focuses on improving drug repurposing through a multisource prompt framework that integrates spatial interaction data and leverages LLMs for reliable drug-target analysis. In domains such as protein analysis and drug design, Protst (Xu et al., 2023) and Drugchat (Liang et al., 2023) employ in-context learning and interactive design to align with user-specific needs.

However, molecular interactions prediction tasks, such as DDIs, still face many challenges. Existing methods typically rely on high-quality fine-tuning data and computationally intensive fine-tuning algorithms (Hu et al., 2021), but the high cost of data acquisition and model training presents significant obstacles. Enhancing the ability of LLMs to predict DDIs under the constraints of limited data and training resources remains a key issue to be addressed.

### 2.3 Prompt engineering for LLM

The framework that combines pre-training and prompts has become a widely recognized best practice in natural language processing, particularly for addressing few-shot and zero-shot tasks (Liu P. et al., 2023). This approach is founded on the principle that LLM possess the capability for in-context learning by leveraging input contexts and instructions (Brown, 2020). Several studies have explored the use of LLM-based approaches for drug design by incorporating various prompting strategies. Li et al. (2024) propose a retrieval-based prompting approach for molecule-caption translation. Liu Y. et al. (2024) introduce MolecularGPT, which provides curated molecular instructions for over 1000 property prediction tasks. Chaves et al. (2024) present TxT-LLM, a method that combines free-text instructions with string representations of molecules throughout different stages of the drug discovery process. However, these methods may not fully capture the complexity of DDIs, which involve various factors including molecular, pharmacological, and clinical considerations.

ICL (Dong et al., 2022) can improve the model’s ability to understand and adapt to different drug combinations by leveraging contextual information from multiple drug-related tasks. Additionally, it allows the model to flexibly adjust its responses based on new data or conditions, such as changes in drug formulations or patient-specific factors, thereby enhancing its predictive capability for DDIs. However, designing more effective ICL prompting paradigms for DDI prediction is an area that requires further study.

## 3 Methods

**FIGURE 1:**
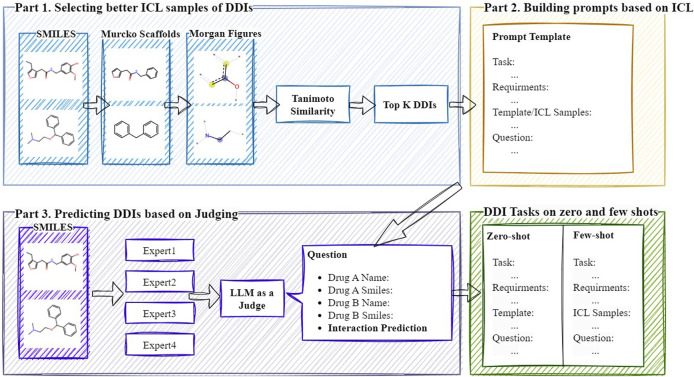
The workflow of DDI-JUDGE.

In this section, we will provide a detailed explanation of the DDI-JUDGE method. This method aims to explore how existing LLMs can be used for DDI prediction, with the overall framework illustrated in Figure 1. The method is primarily divided into three parts: 1) Selecting ICL samples, 2) Building prompts based on ICL, and 3) Generating an LLM-based discriminator to integrate multi-model results. First, DDI-JUDGE leverages drug similarity to select optimal prompt samples, performing positive sample selection and hard negative sample mining. Next, based on the selected prompt samples, we construct prompt templates specifically designed for DDI prediction. Finally, we use GPT to generate an LLM-based discriminator, which scores the predictions of multiple LLMs and integrates the results based on the scores.

### 3.1 Selecting better ICL samples of DDIs

ICL is a prompting paradigm applied to LLMs, which enhances the capabilities of LLM by using a small number of demonstration prompts. In DDI prediction, we need to study how to find more suitable prompt examples. In order to better select prompt examples, we propose an ICL positive and negative samples selection method for DDI based on drug similarity calculation. Three widely used similarity measures include Tanimoto similarity (Tanimoto, 1958), Cosine similarity, and Dice similarity. Tanimoto To more effectively assess the similarity of drug feature vectors, we examine the variations in the outcomes produced by these methods. In drug similarity calculations, Dice similarity emphasizes shared structural features, making it suitable for identifying common substructures. Cosine similarity focuses on the angular relationship of feature vectors, ideal for analyzing high-dimensional molecular data. Tanimoto similarity balances shared and unique molecular features, making it particularly effective for comparing molecular fingerprints in chem-informatics. Let $x=\left(x_{1},x_{2},\ldots ,x_{n}\right)$ and $y=\left(y_{1},y_{2},\ldots ,y_{n}\right)$ represent the binary molecular fingerprints of two drugs got from Rdkit (RDKit, 2013), where each element indicates the presence or absence of a specific substructure. The Tanimoto similarity is defined as shown in Equations 1:

$${Sim}_{T}\left(x,y\right)=\frac{x\cdot y}{{\left\|x\right\|}^{2}\times {\left\|y\right\|}^{2}}=\frac{\sum x_{i}y_{i}}{\sqrt{\sum x_{i}^{2}}+\sqrt{\sum y_{i}^{2}}-\sum x_{i}y_{i}}$$ (1)

where x·y denotes the dot product (inner product) of x and y, calculated as $\sum x_{i}y_{i}$ , i.e., the summation of element-wise products. ${\left\|x\right\|}^{2}$ and ${\left\|y\right\|}^{2}$ represent the squared norms of vectors x and y, respectively. Here, the numerator represents the number of shared substructures, while the denominator captures the total number of unique substructures across both molecules.

In addition to Tanimoto similarity, we also evaluate Cosine similarity, which captures the angular distance between vectors of drugs. For the molecular fingerprints of two drugs x and y, the similarity can be calculated as shown in Equations 2:

$${Sim}_{C}\left(x,y\right)=\frac{x\cdot y}{{\left\|x\right\|}^{2}\times {\left\|y\right\|}^{2}}=\frac{\sum x_{i}y_{i}}{\sqrt{\sum x_{i}^{2}}\sqrt{\sum y_{i}^{2}}}$$ (2)

Here, this formulation captures the relative orientation between molecular feature vectors. Further, Dice similarity can be calculated as shown in Equations 3:

$${Sim}_{D}\left(x,y\right)=\frac{2\sum x_{i}y_{i}}{\sum x_{i}^{2}+\sum y_{i}^{2}}$$ (3)

where the dice similarity ranges from 0 to 1. In addition to traditional fingerprint-based similarity measures, we further explore two additional categories of similarity metrics to enhance the selection of ICL examples: graph-based similarity and embedding-based similarity. To explore structural similarity at the graph level, we utilize the Weisfeiler-Lehman graph kernel. Let $G_{1}$ and $G_{2}$ denote two molecular graphs, and let $\emptyset \left(G\right)$ be the mapping of a graph to a high-dimensional feature space based on its structural patterns. The similarity between two embeddings is computed using their dot product, as defined in Equations 4:

$${Sim}_{G}\left(G_{1},G_{2}\right)=\langle \emptyset \left(G_{1}\right),\emptyset \left(G_{2}\right)\rangle$$ (4)

where $\langle *\rangle$ represents the dot product. This approach captures topological information beyond atom-level fingerprints, enabling graph-level matching when selecting prompts based on molecular structure.

For embedding-based similarity, we leverage pretrained deep learning models to extract SMILES-based embeddings. Given a pair of drugs x and y, their corresponding embedding vectors are denoted as $e_{x}$ and $e_{y}$ . The cosine similarity between the embedding vectors is used to compute their similarity, as shown in Equations 5:

$${Sim}_{E}\left(x,y\right)=\frac{e_{x}\cdot e_{y}}{{\left\|e_{x}\right\|}^{2}\times {\left\|e_{y}\right\|}^{2}}$$ (5)

This embedding-based metric captures both structural and functional properties encoded during pretraining, providing a complementary perspective to symbolic similarity. In our implementation, the embeddings are generated from SMILES sequences using the pretrained MolBERT (Fabian et al., 2020) model. Finally, We utilize the Tanimoto similarity based on 2048-bit Morgan fingerprints (Morgan, 1965) with a radius of two to calculate molecular scaffold similarity. The similarity score for each candidate drug pair is calculated as the product of the similarity scores of the two drugs. Among the known positive DDI samples, we identify the top-k most similar molecular SMILES pairs to construct positive sample prompts. Similarly, for negative sample prompts, we select the top-k most similar SMILES DDI pairs.

### 3.2 Building prompts based on ICL

In recent advances in language models, ICL has emerged as a method to enable models to learn tasks without explicit fine-tuning. ICL achieves this by providing examples within the input, allowing the model to understand the task through context and generate accurate outputs. Based on filtered positive and negative sample examples, we constructed prompts for DDI prediction, which are categorized into zero-shot and few-shot scenarios.

**FIGURE 2:**
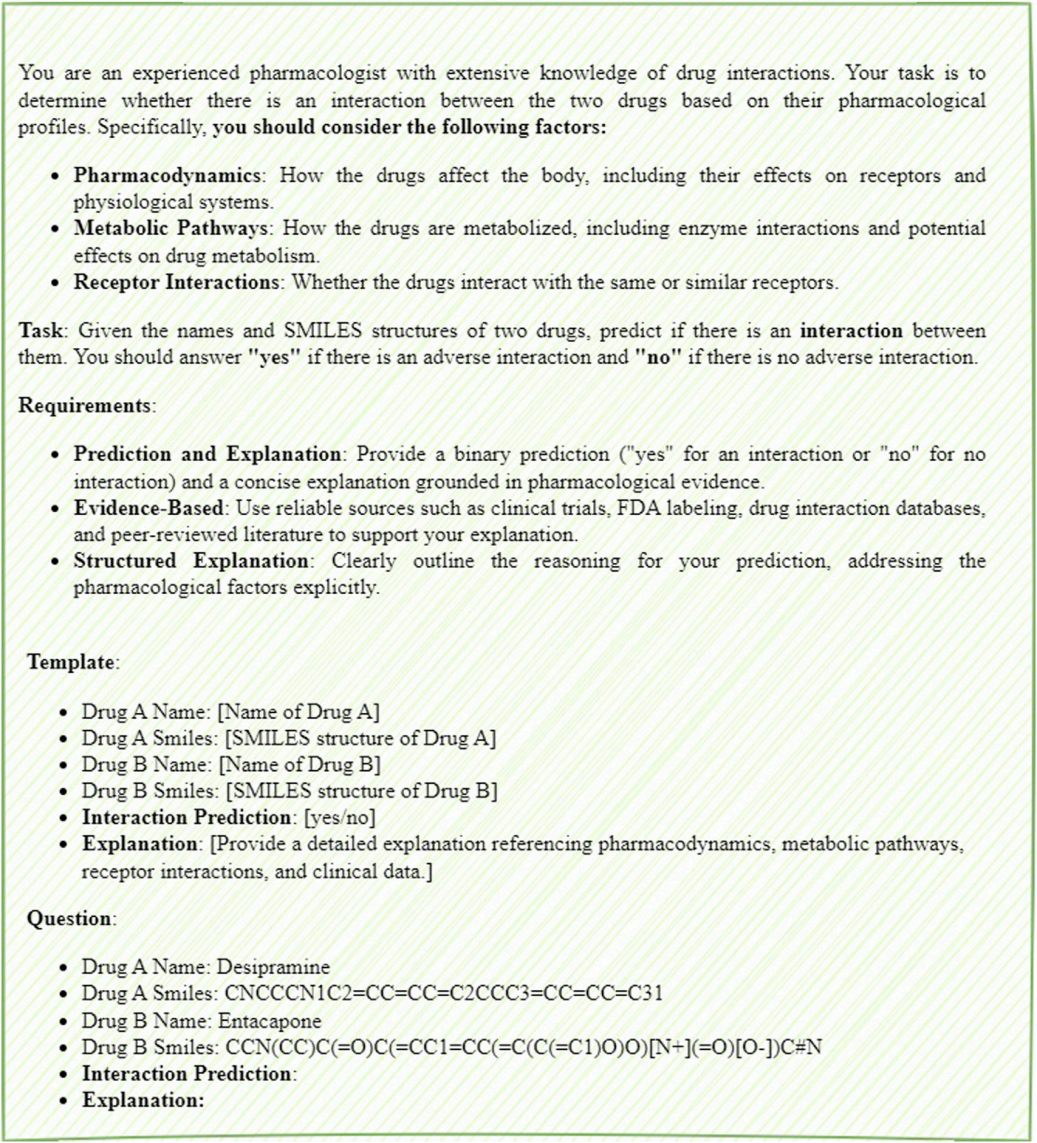
The zero-shot prompt of DDIs prediction.

**FIGURE 3:**
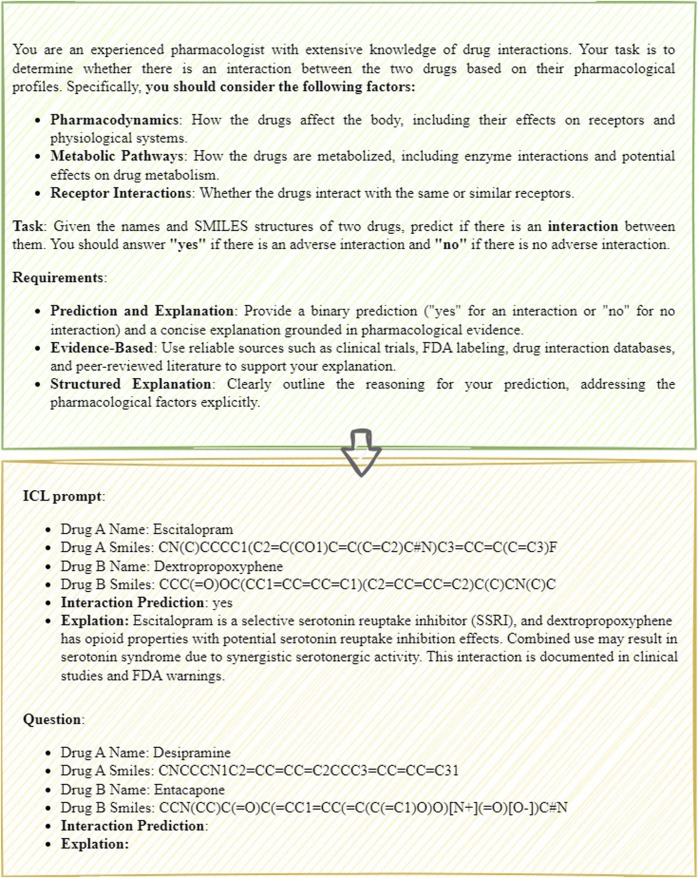
The few-shot prompt of DDIs prediction.

In the zero-shot scenario, the model makes predictions based purely on its pre-trained knowledge, without relying on specific examples. This approach is suited for predicting interactions between novel or previously unseen drug combinations as shown in Figure 2. In contrast, the few-shot scenario provides a small set of examples to help guide the model’s predictions, particularly when limited data or related examples are available as shown in Figure 3. The prompt follows a structured format, consisting of several key components: input requirements, prediction task, consideration factors, and examples. The input requirements specify the drug names and their corresponding SMILES structures. The prediction task involves predicting whether an interaction exists between the two drugs, with the outcome being “yes” or “no.” Consideration factors include an analysis of pharmacodynamics, metabolic pathways, receptor interactions, and relevant clinical data, including FDA labels and peer-reviewed literature. Finally, the examples section provides a practical demonstration of the input format and expected prediction output, ensuring clarity in applying the model.

### 3.3 Predicting DDIs based on judging

**FIGURE 4:**
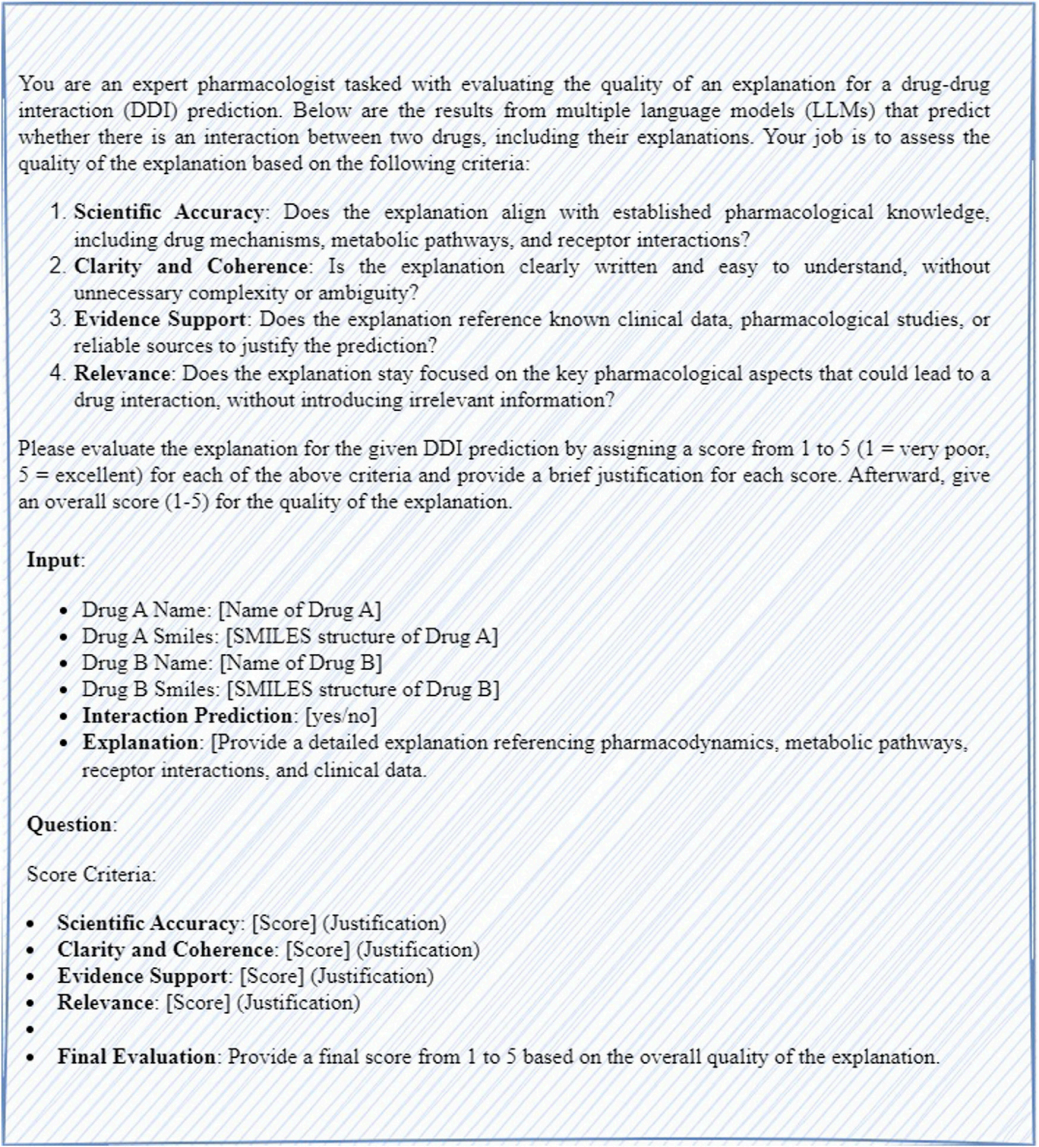
The prompt of the DDIs prediction judge.

In this study, we use GPT-4 as a judge to evaluate DDIs prediction generated by multiple LLMs. The discriminator assesses the quality of the explanations provided for each DDI prediction based on four key criteria: scientific accuracy, clarity and coherence, evidence support, and relevance. A detailed prompt is designed for both zero-shot and few-shot scenarios as shown in Figure 4. In the zero-shot scenario, the prompt clearly outlines the evaluation criteria and instructions for GPT to assess each prediction and explanation. In the few-shot scenario, the prompt includes several examples of high-quality evaluations to help the model understand how to assign scores. Each explanation is scored on a scale from one to five for each criterion, and an overall score is assigned based on the evaluation. After scoring the results from all models, the predictions are combined using a weighted fusion approach, where each model’s score is multiplied by a predetermined weight reflecting its reliability or performance, and the weighted scores are summed to generate the final DDI prediction.

After scoring the results from all models, the predictions are combined using a weighted fusion approach, where the weight $w_{i}$ ​ for each model *i* is determined by the score given by the discriminator to that model’s output $S_{model}$ is calculated as shown in Equations 6:

$$S_{final}=\sum_{i=1}^{N}w_{i}S_{model}$$ (6)

Compared to other ensemble learning techniques such as stacking or boosting, we adopt a weighted fusion strategy to maintain a streamlined, inference-oriented workflow. This approach eliminates the need to train extra models and fits well with LLM workflows that rely mainly on inference rather than supervised training.

## 4 Results

### 4.1 Datasets

In the paper, we use the Luo’s dataset (Luo et al., 2017) contains the following information, as shown in Table 1. It integrates information from multiple authoritative biomedical sources. Specifically, drug-related information was obtained from DrugBank 3.0 (Craig et al., 2010), protein data from Human Protein Reference Database (Suraj et al., 2004), disease associations from Comparative Toxicogenomics Database (Mattingly et al., 2003), and side-effect information from SIDER (Michael et al., 2016). These heterogeneous entities—including drugs, proteins, diseases, and side effects—were incorporated into a unified heterogeneous network. The dataset includes 12,015 nodes (708 drugs, 1,512 proteins, 5,603 diseases, and 4,191 side effects) and over 1.89 million edges, with 10,036 known drug-drug interactions. This large-scale, multi-relational structure allows for comprehensive modeling of biomedical interactions.

**TABLE 1:** The detail of LUO’s datasets.

Node types	Num	Edge types	Num
Drug	708	Drug-drug	10,036
Protein	1,512	Drug-protein	1,923
Disease	5,603	Protein-protein	7,363
Side Effect	4,192	Drug-Disease	199,214
		Protein-Disease	1,596,745

In our study, we employ cross-validation to evaluate the effectiveness of our proposed method. Specifically, we use a 10-fold cross-validation approach. The dataset is randomly partitioned into ten subsets, from which nine subsets are used for training and the remaining one for testing. This process is repeated ten times, with each subset serving as the test set once. The final performance result is computed as the average of the outcomes from all ten iterations. The final performance is reported as the average of the results across all ten folds, which helps reduce variance due to random partitioning and enables reliable comparison of different models. In the zero-shot scenario, the model is directly tested using the test set. In the few-shot scenario, positive and negative samples are selected from the training set for use in context learning prompts.

### 4.2 Evaluation criteria

DDI prediction is a classification task where the outcomes are categorized into four types: true positive (TP), false positive (FP), true negative (TN), and false negative (FN). Based on these classifications, AUPR (Area Under the Precision-Recall Curve) and AUC (Area Under the ROC Curve) are widely used evaluation metrics. 1) AUC assesses the model’s ability to rank true DDIs higher than non-DDIs across all possible thresholds. It reflects the trade-off between the true positive rate (TPR = TP/(TP + FN)) and the false positive rate (FPR = FP/(FP + TN)), providing a comprehensive view of classification performance. 2) AUPR focuses on the balance between precision (TP/(TP + FP)) and recall (TP/(TP + FN)), which is particularly informative in imbalanced datasets such as DDI, where positive examples are much rarer than negatives.

In summary, higher AUC and AUPR values indicate that the model is better at identifying true interactions while minimizing false positives, which is critical in real-world pharmaceutical applications where missing or wrongly predicting DDIs can have serious consequences.

### 4.3 Comparison models

Given our focus on zero-shot and few-shot scenarios, we primarily selected models based on LLMs. These models include GPT-4 (Achiam et al., 2023), GPT-3.5 (Brown, 2020), Davinci-003, and llama 2 (Touvron et al., 2023b). In addition to these well-established models, we also included more recent state-of-the-art models such as llama 3 (Dubey et al., 2024), GPT-4o, DeepSeek V3 (Liu A. et al., 2024), and Claude 3.5 (Bae et al., 2024). All these models leverage the Transformer architecture, utilizing self-attention mechanisms and large-scale pretraining to achieve efficient generation and understanding of natural language processing tasks through deep learning techniques. Specifically, GPT-4 and GPT-3.5 are known for their advanced reasoning and language understanding capabilities, while Davinci-003 provides a robust foundation for few-shot learning. The inclusion of GPT-4o, DeepSeek V3, and Claude 3.5 ensures that our benchmark is up-to-date with the latest advancements in the field.

### 4.4 Comparison experiments

First, to analyze the impact of drug similarity on the selection of positive and negative drug pairs as ICL prompts in DDI-JUDGE, we discuss the effects of Cosine similarity, Dice similarity, and Tanimoto similarity on the final results. As shown in Table 2, Tanimoto similarity achieves the best performance, although the differences among the three are minimal. Tanimoto similarity is particularly suitable for drug similarity calculation as it effectively balances shared and unique features, accurately capturing the chemical relationships between drug molecules. To provide a more comprehensive discussion on the role of similarity metrics in DDI-JUDGE, we further evaluate two additional approaches: graph-based similarity and embedding-based similarity. Specifically, we apply the WL graph kernel to compute graph similarity, and use SMILES-based embeddings generated by MolBERT to measure embedding similarity. According to Table 2, the embedding-based method achieves the best overall performance, with an AUC of 0.794 and AUPR of 0.815, surpassing all other methods. The graph-based approach also performs competitively, with results close to those of Tanimoto, indicating that incorporating molecular topology can be beneficial. These results suggest that embedding-based similarity is particularly effective for capturing deeper structural and semantic information.

**TABLE 2:** The results of three similarity measure.

Similarity	AUC	AUPR
Cosine	0.763	0.799
Dice	0.779	0.787
Tanimoto	0.788	0.801
Graph-based	0.785	0.798
Embedding-based	0.794	0.815

Overall, while Tanimoto similarity remains the most efficient and effective choice in our method, graph-based and embedding-based similarities present valuable alternatives that can be further explored or integrated in future improvements.

We mainly conducted experiments in two scenarios: zero-shot and few-shot. Zero-shot refers to the model predicting DDIs without any prior training examples or specific task prompts, relying solely on its pre-trained knowledge. Few-shot involves providing the model with a small number of examples, such as known interactions between drugs, to help it understand the task requirements before making predictions.

The experimental results under the zero-shot setting, as shown in Table 3, reveal that DDI-JUDGE demonstrates the best performance among all models, achieving the highest AUC (0.642) and AUPR (0.629). GPT-4o and DeepSeek V3 also perform well with AUC and AUPR values of 0.585/0.557 and 0.603/0.581, respectively. Llama two exhibits relatively weak performance, with an AUC of 0.382 and an AUPR of 0.400.

**TABLE 3:** The experimental results on the zero-shot scenario.

Methods	AUC	AUPR
GPT-4o	0.585	0.603
GPT-4	0.557	0.581
GPT-3.5	0.521	0.535
Davinci-003	0.443	0.416
llama 2	0.382	0.400
llama 3	0.573	0.551
Claude 3.5	0.536	0.577
DeepSeekV3	0.541	0.589
DDI-JUDGE	0.642	0.629

In the few-shot setting, as presented in Table 4, DDI-JUDGE once again achieves the highest performance, with an AUC of 0.788 and an AUPR of 0.801, showcasing its robustness when provided with a few examples. Davinci-003 and llama two show comparatively weaker performance, with AUC/AUPR values of 0.525/0.553 and 0.417/0.488, respectively. The results demonstrate that DDI-JUDGE effectively leverages few-shot examples to maintain its superior predictive capabilities.

**TABLE 4:** The experimental results on the few-shot scenario.

Methods	AUC	AUPR
GPT-4o	0.681	0.643
GPT-4	0.656	0.637
GPT-3.5	0.632	0.622
Davinci-003	0.525	0.553
llama 2	0.417	0.488
llama 3	0.631	0.658
Claude 3.5	0.647	0.626
DeepSeekV3	0.679	0.631
DDI-JUDGE	0.788	0.801

Comparing the two settings, it is evident that all models benefit from the few-shot scenario, as providing a small number of examples improves their performance. DDI-JUDGE shows significant improvement, with its AUC increasing from 0.642 (zero-shot) to 0.768 (few-shot) and its AUPR rising from 0.629 to 0.760. Overall, few-shot learning enhances the models' predictive performance, with DDI-JUDGE maintaining its leading position across both settings.

### 4.5 The impact of the number of ICL prompt samples

Next, we discussed the impact of different numbers of ICL prompt samples on the predictive performance, as shown in Table 5. As the number of ICL prompt samples increases, DDI-JUDGE’s performance improves significantly. Starting from zero-shot, the AUC and AUPR steadily rise as more prompt samples are provided, with the best performance achieved when eight samples are used. These results suggest that increasing the number of ICL prompt samples provides more contextual information, allowing the model to better understand the task and make more accurate predictions.

**TABLE 5:** The results on different numbers of ICL prompt samples.

Methods	AUC	AUPR
DDI-JUDGE (zero-shot)	0.642	0.629
DDI-JUDGE (n = 1)	0.662	0.671
DDI-JUDGE (n = 2)	0.679	0.694
DDI-JUDGE (n = 4)	0.731	0.752
DDI-JUDGE (n = 8)	0.788	0.801

### 4.6 Case study

Nowadays, an increasing number of studies are exploring whether methods can be directly translated into practical improvements in real-world drug discovery and are conducting relevant experiments (Zhao et al., 2022; Wang et al., 2025a; Wang et al., 2025b; Zhao et al., 2024; Zhao et al., 2025). To demonstrate the capability of our method in addressing real-world drug discovery issues, we conducted experiments and identified several DDIs that are not present in the DrugBank database.

1) When Rivaroxaban is used concomitantly with Dihydroxyaluminum Sodium Carbonate, the anticoagulant effect of Rivaroxaban may be compromised due to the potential for increased gastrointestinal bleeding in patients with gastroduodenal ulcers (Goldhaber, 2020).
2) When romidepsin is used concomitantly with quinidine, the risk or severity of QT interval prolongation may be increased. Romidepsin, a histone deacetylase inhibitor, is employed in the treatment of certain types of lymphoma; quinidine is an antiarrhythmic agent (Abu Rmilah et al., 2020).
3) Simvastatin is a cholesterol-lowering drug that works by inhibiting the enzyme HMG-CoA reductase. Fluconazole is a triazole antifungal agent. Studies have shown that the concurrent use of these two drugs may increase the risk of myopathy or rhabdomyolysis (Molden et al., 2008).

The case studies demonstrate the capacity of DDI-JUDGE to identify novel DDIs. Consequently, DDI-JUDGE exerts a beneficial influence on the design and development process of new drugs.

## 5 Conclusion

In this paper, we propose an LLM-based method for DDI prediction, which is achieved through the integration of judging and ICL prompts. The proposed method outperforms existing LLM approaches, demonstrating the potential of LLMs for predicting complex relationships in drug molecules.

First, we propose a novel ICL prompt paradigm for DDI prediction. This approach selects high-similarity samples as positive and negative prompts, enabling the LLM to effectively learn and generalize knowledge. Additionally, we introduce an ICL-based prompt template that organizes structured prompts, including input requirements, prediction tasks, relevant factors, and examples. By leveraging the pre-trained knowledge and contextual understanding of LLMs, this template enhances DDI prediction capabilities. Finally, we employ GPT-4 as a discriminator to assess the predictions of multiple LLMs based on scientific accuracy, clarity, evidence support, and relevance. These individual results are then combined through a weighted fusion method to improve prediction accuracy.

In addition, this study emphasizes zero-shot and few-shot prompting scenarios, which reflect the practical challenges of real-world DDI prediction, where labeled data are often scarce. As shown in our analysis, performance improves as the number of prompt examples increases, including the one-shot setting. Many-shot prompting, although potentially beneficial, was not explored further due to input length limitations and diminishing marginal gains. These findings highlight that zero-shot and few-shot prompting offer an effective and scalable approach to DDI prediction in settings with limited labeled data.

The method currently has the following limitations: While it explores the potential of applying LLMs to DDI prediction, there is still a lack of domain-specific drug knowledge. For example, GPT-4, as a discriminator, may introduce potential biases due to its inability to fully understand domain-specific knowledge and scientific context. Future work will incorporate more drug-related data and perform fine-tuning to further optimize the performance.

## Data Availability

The original contributions presented in the study are included in the article/supplementary material, further inquiries can be directed to the corresponding authors.
